# Engineering the Calvin–Benson–Bassham cycle and hydrogen utilization pathway of *Ralstonia eutropha* for improved autotrophic growth and polyhydroxybutyrate production

**DOI:** 10.1186/s12934-020-01494-y

**Published:** 2020-12-11

**Authors:** Zhongkang Li, Xiuqing Xin, Bin Xiong, Dongdong Zhao, Xueli Zhang, Changhao Bi

**Affiliations:** 1 Tianjin Institute of Industrial Biotechnology, Chinese Academy of Sciences, Tianjin, China; 2 Key Laboratory of Systems Microbial Biotechnology, Chinese Academy of Sciences, Tianjin, China

**Keywords:** Calvin–Benson–Bassham cycle, Carbon fixation pathway, *Ralstonia eutropha*, Polyhydroxybutyrate

## Abstract

**Background:**

CO_2_ is fixed by all living organisms with an autotrophic metabolism, among which the Calvin–Benson–Bassham (CBB) cycle is the most important and widespread carbon fixation pathway. Thus, studying and engineering the CBB cycle with the associated energy providing pathways to increase the CO_2_ fixation efficiency of cells is an important subject of biological research with significant application potential.

**Results:**

In this work, the autotrophic microbe *Ralstonia eutropha (Cupriavidus necator*) was selected as a research platform for CBB cycle optimization engineering. By knocking out either CBB operon genes on the operon or mega-plasmid of *R. eutropha*, we found that both CBB operons were active and contributed almost equally to the carbon fixation process. With similar knock-out experiments, we found both soluble and membrane-bound hydrogenases (SH and MBH), belonging to the energy providing hydrogenase module, were functional during autotrophic growth of *R. eutropha.* SH played a more significant role. By introducing a heterologous cyanobacterial RuBisCO with the endogenous GroES/EL chaperone system(A quality control systems for proteins consisting of molecular chaperones and proteases, which prevent protein aggregation by either refolding or degrading misfolded proteins) and RbcX(A chaperone in the folding of Rubisco), the culture OD_600_ of engineered strain increased 89.2% after 72 h of autotrophic growth, although the difference was decreased at 96 h, indicating cyanobacterial RuBisCO with a higher activity was functional in *R. eutropha* and lead to improved growth in comparison to the host specific enzyme. Meanwhile, expression of hydrogenases was optimized by modulating the expression of MBH and SH, which could further increase the *R. eutropha* H16 culture OD_600_ to 93.4% at 72 h. Moreover, the autotrophic yield of its major industrially relevant product, polyhydroxybutyrate (PHB), was increased by 99.7%.

**Conclusions:**

To our best knowledge, this is the first report of successfully engineering the CBB pathway and hydrogenases of *R. eutropha* for improved activity, and is one of only a few cases where the efficiency of CO_2_ assimilation pathway was improved. Our work demonstrates that *R. eutropha* is a useful platform for studying and engineering the CBB for applications.

## Background

The stability and balance of the biosphere is maintained by the flow of carbon back and forth between life forms and the environment through various biogeochemical cycles [1]. According to information from the International Energy Agency, the amount of carbon dioxide released in 2007 was 28.8Gt, which is expected to increase to 40.3Gt by 2030 and 50Gt by 2050, if no appropriate measures are taken [2]. Therefore, we urgently need to develop technologies for carbon recycling or increase the capacity of natural organisms for CO_2_ fixation.

The Calvin–Benson–Bassham (CBB) cycle, which utilizes the CO_2_ fixation enzyme ribulose-1,5-bisphosphate carboxylase/oxygenase (RuBisCO), is a key biological pathway for converting atmospheric CO2 to organic matter. It is of great significance to the global carbon cycle and crop production, and widely distributed in most autotrophic organisms including plants, algae, cyanobacteria, as well as other photo- and chemoautotrophic bacteria [3]. Apart from the CBB cycle, five other carbon fixation pathways have been discovered in nature, among which the reductive acetyl-CoA pathway has the highest CO_2_ fixation efficiency under anaerobic conditions, whereby 2 mol CO_2_ are fixed into 1 mol of acetyl-coA using 1 mol ATP and 4 mol NAD(P)H [4]. CBB is more energy intensive, requiring 9 mol ATP and 6 mol NAD(P)H for the fixation of 3 mol CO_2_, but is not sensitive to oxygen and is widely distributed in higher plants, algae and cyanobacteria, which makes improving its efficiency a highly promising prospect [4].

Most research and engineering of the CBB cycle has focused on improving the reaction efficiency of carboxylation by the enzyme RuBisCO, which can be classified into four groups. While all known forms of RuBisCO are composed of catalytic large subunit dimers, the difference lies in different numbers of catalytic larger dimers [5]. It was reported that the assembly of the large dimer requires the synergistic effects of GroES/EL, and assembly of the final RuBisCO octamer comprising the large subunit dimer with RubS requires the RubX chaperone, while the assembly of fully formed RuBisCO does not require the participation of RubX [6, 7]. There are a number of strategies to improve the CO_2_ fixation efficiency, including adaptive evolution of RuBisCO catalytic subunits and the promoter of the CBB operon [8], co-expression of auxiliary pathways, and heterologous introduction of highly catalytic RuBisCO. For example, Lin et al. introduced RuBisCO and RubS from the cyanobacterium *Synechococcus elongatus* PCC 7942 into tobacco, and achieved an improvement of carbon fixation efficiency. In addition, it was verified that the introduction of a CO_2_-concentrating mechanism (CCM) could improve the efficiency of CO_2_ fixation [9]. However, there are very few successful cases, probably due to limited improvement of RuBisCO by adaptive evolution, and the long experimental cycles needed for research in plants.

*Ralstonia eutropha* H16 *(Cupriavidus necator*) is a Gram-negative facultatively chemoautotrophic bacterium, which cannot only use fructose, gluconic acid and other organic carbon sources for heterotrophic growth, but also uses CO_2_ and H_2_ for autotrophic growth in the presence of O_2_. Different from most chemoautotrophic bacteria, *R. eutropha* H16 utilizes the CBB cycle for carbon fixation, which is very similar to that of eukaryotes such as plants. Due to its much shorter generation time compared with plants, *R. eutropha* is a potential platform for optimization of the CBB cycle. In addition, this bacterium has been successfully metabolically engineered to produce various chemicals, such as ethanol [10], isobutanol [11], fatty acids, hydrogen [12] and alkanes [13], which suggests that *R. eutropha* H16 has great potential for development of various biotechnological applications using CO_2_ sequestration.

With the deciphering of its genome sequence [14, 15], the genes involved in autotrophic growth of *R. eutropha* were identified in silico, including four hydrogenases encoded on the large plasmid, and two copies of the Calvin–Benson–Bassham (CBB cycle) operon, one on chromosome 2 and one on the large plasmid. The CBB operon of *R. eutropha* on chromosome includes 14 genes, which are cbbR, L, S, X, Y, E, F, P, T, Z, G, K, A and B. These genes and their coded enzyme of CBB operon is listed in Additional file 1: Table S3. The copy on mega plasmid has all genes except cbbB. Hydrogenases catalyze the oxidation of hydrogen to form 2e^−^ and 2[H]^+^. The four hydrogenases of *R. eutropha* H16 are membrane-bound hydrogenase (MBH), soluble hydrogenase (SH), regulatory hydrogenase (RH) and a fourth NiFe hydrogenase (Hyd4) [16]. All of them are oxygen-resistant members of the [NiFe]-hydrogenase family. The two hydrogenases MBH and SH, which have distinct functions, have been purified and analyzed [17]. MBH is composed of HoxK and HoxG structural subunits, which are anchored to the membrane by HoxZ. This complex delivers electrons to B-type cytochrome, and further to the electron transport chain to provide energy for *R. eutropha* H16 cells. Protons are delivered to periplasm [18]. SH is heterotetrameric complex composed of HoxH, Y, F, and U subunits, which delivers protons and electrons to NAD^+^ to synthesize NADH for cell growth and biosynthetic reactions. Its maturation requires a series of accessory proteins to assist its complex assembly. The third hydrogenase, RH, is a H_2_-sensing regulatory [NiFe]-hydrogenase consisting of HoxB, C and J subunits. It acts as a signal protein that controls the functional expression of MBH and SH, independent of intracellular energy and NADH status [17]. Hy4 hydrogenase is one of the least well-characterized hydrogenases. It consists of the two structural subunits PHG064 and PHG065, as well as a number of auxiliary proteins, including hypF3, hypC2, hypD2, hypE2, hypA3, and hypB3, whose physiological roles and expression mechanisms are still not fully known [16]. On the other hand, the CBB operon on the *R. eutropha* chromosome is much better understood. It encodes an inversely arranged lysine family transcriptional regulator protein CbbR, and a polycistronic CBB expression cassette. Moreover, the two copies of the CBB operon have high homology. The lysine family regulatory protein located on the megaplasmid is not complete, but the CbbR expressed from the chromosome still regulates the expression of the CBB operon on the megaplasmid [19].

Previous studies provided a basis for engineering the autotrophic metabolic pathways of *R. eutropha.* In this study *R. eutropha* H16 was selected for research and engineering due to its potential to be developed as an efficient and convenient platform for optimization of the CBB cycle, as well as an efficient CO_2_-sequestrating cell factory for various biotechnological applications.

## Results and discussion

### Determination of the contribution of the CBB operons and hydrogenases to autotrophic growth of *R. eutrophic* H16

The CBB cycle is an expensive metabolic pathway that consumes large amounts of energy and reducing equivalents. The production of 1 mol of 3-PG from 3 mol of CO_2_ requires 9 mol of ATP and 6 mol of NADPH. In *R. eutropha* H16 cells, the hydrogenase systems are employed to provide both the energy and the reducing equivalents for the CBB cycle [4]. The hydrogenases of *R. eutropha* H16 are insensitive to oxygen, which is rather unusual. They catalyze the oxidation of molecular hydrogen into protons and electrons. Electrons are then transferred to membrane-bound or cytoplasmic electron carriers with oxygen as the terminal electron acceptor [20]. This process either generates the proton electromotive force for ATP production or provides NADH. [21], or is used for the regeneration NADH through the cytoplasmic electron transport chain [22]. There are few studies on the three main hydrogenases of the autotrophic system of *R. eutropha* H16, i.e. SH, MBH and RH, and their individual contribution to carbon fixation and autotrophic growth is unknown. It was reported that the CBB operon on chromosome 2 of *R. eutropha* H16 contains 13 CBB coding sequences, along with a *cbbR* gene on the negative strand, while the CBB operon on the megaplasmid of *R. eutropha* H16 consists of 12 CBB coding sequences with a deficient *cbbR* gene [19]. The CbbR expressed from chromosome 2 was considered to control the expression of both CBB operons [19]. Here, we estimated which hydrogenase or CBB operon is more important for autotrophic growth by constructing corresponding knockout strains.

The knockout strains and strains carrying overexpression plasmids were subjected to autotrophic fermentation, which is performed in minimal medium supplemented with only a gas mixture comprising H_2_, CO_2_, and O_2_ at a volume ratio of 7:1:1, but no organic substrate is provided. According to non-systematic experiments, the growth of *R. eutropha* could reach 14 of OD 600 in the autotrophic fermentation at 369 h (data not shown). Under such conditions, CO_2_ was the only carbon source for the synthesis of cellular building blocks, and the cell growth efficiency was assumed to be directly correlated with the carbon fixation efficiency. The strains are summarized in Additional file 1: Table S1, and their autotrophic growth phenotype is illustrated in Additional file 1: Figure S1. The results indicated that a double knockout of both MBH and SH hydrogenases completely eliminated the autotrophic growth capacity of *R. eutropha* H6, while a single deletion of either MBH or SH only partly affected the autotrophic growth. Based on the growth profile, the deletion of SH had a more significant impact (Additional file 1: Figure S1A). Thus, while both hydrogenases were functional during autotrophic growth of *R. eutropha* H6 and contributed to the cell metabolism, the results indicated that SH probably played a more significant role.

In the case of the CBB enzymes, both single deletion of RuC (RuBisCO operon on Chromosome 2) or RuP (RuBisCO operon on the megaplasmid) affected the autotrophic growth efficiency, and the growth decrease was similar for both operons (Additional file 1: Figure S1B), which indicated that both CBB operons were active and contributed almost equally to the carbon fixation process.

To determine if there was a possible polar effect of the knockout experiments, complementation experiments were performed to see if we could restore the autotrophic growth capacity of the double knock out strains of RuBisCO or hydrogenases via plasmid-based expression of the knocked-out genes. The complemented strain H16ΔRuPΔRuC(pRrub) carrying plasmid pRrub Additional file 1: Table S1, expressing rubisco units of *R. eutropha,* recovered its autotrophic growth ability, indicating that there was no polar effect of the RuBisCO knockouts (Additional file 1: Figure S1C). However, the growth of the complemented strain was not as good as the wild type strain, which we considered as an acceptable variation in the gas fermentation process. In addition, the complementation of the two hydrogenases in strain H16ΔMBHΔSH was not successful. Considering the difficulties of a manipulating large number of genes in *R. eutropha*, the failed complementation experiment does not necessarily indicate a polar effect for the knockout.

In summary, we determined that both the SH and MBH hydrogenases contribute to the autotrophic growth of *R. eutropha* with SH significantly more important than MBH, and both CBB operons are active in the carbon fixation process.

### Engineering the CBB cycle for improved autotrophic growth of *R. eutropha*

**Fig. 1:**
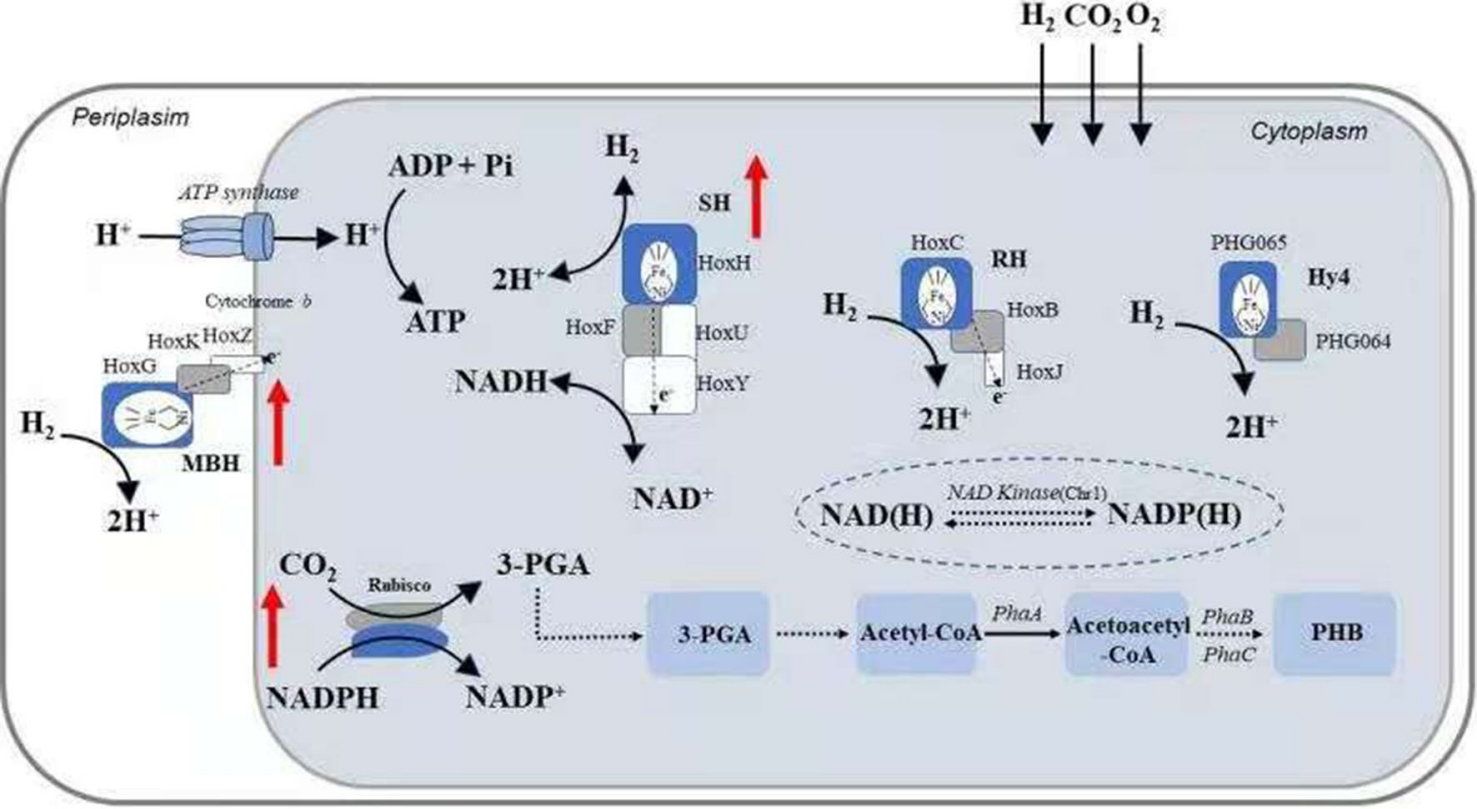
Overview of the autotrophic metabolic pathways and engineering strategy of a *R. eutropha* H16 microbial cell factory to improve autotrophic growth and PHB production. The autotrophic metabolism of *R. eutropha* H16 is based on the CBB cycle and hydrogenases for energy supply. The red arrows indicate focuses of the engineering strategy. PhaA: β-ketothiolase; PhaB: NADPH-dependent acetoacetyl-CoA reductase; phaC: PHB synthase; MBH: membrane-bound hydrogenase; SH: soluble hydrogenase; RH: regulatory hydrogenase; Hyd4: NiFe hydrogenase

The autotrophic metabolism of *R. eutropha* H16 constitutes of the CBB cycle and hydrogenases. In the CBB cycle, RuBisCO catalyzes the carboxylation reaction converting ribulose-1,5-diphosphate and CO_2_ to generate 2 molecules of 3-phosphoglyceric acid for the synthesis of organic carbon compounds. The efficiency of RuBisCO is low and it is considered the speed-limiting step of the CBB cycle (Fig. 1) [9]. Therefore, we intended to improve the carbon fixation efficiency of *R. eutropha* H16 by increasing the efficiency of its RuBisCO enzyme. The RuBisCO with the highest reported efficiency is that from the cyanobacterium *Synechococcus* sp. PCC 7002 [8]. Due to the complex structure of RuBisCO, its heterologous folding and maturation might not be ideal and require chaperones or accessory proteins. Previous studies have shown that the GroES/EL chaperone system of *E. coli* is of great significance for the folding of heterologous RuBisCO proteins [23]. Therefore, in this study we attempted to overexpress the endogenous RuBisCO from *R. eutropha* or the heterologous cyanobacterial RuBisCO, coupled with various chaperone systems to find an optimal strategy for increasing the CO_2_ fixation capacity.

**Fig. 2:**
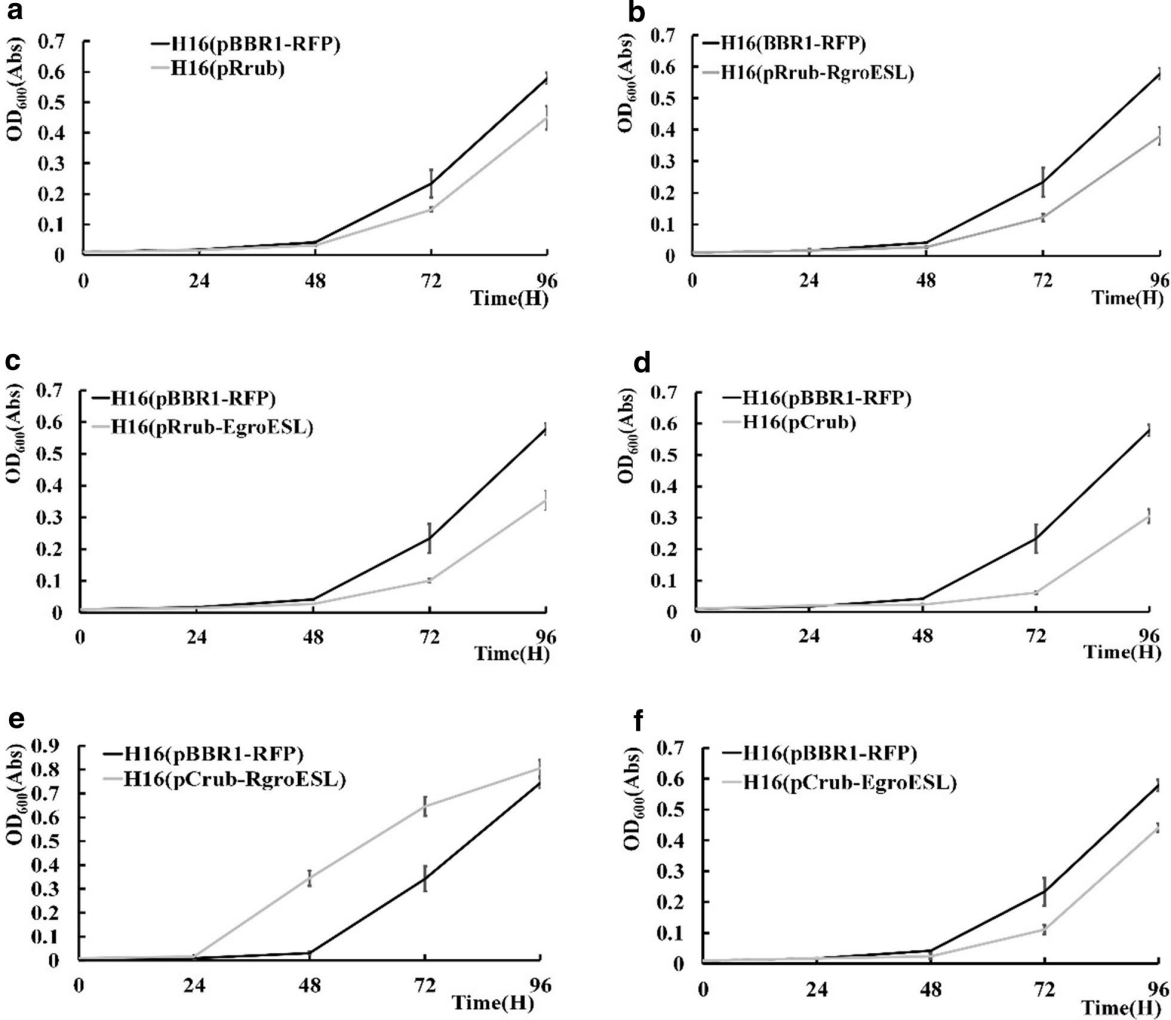
Optimization of RuBisCO expression in *R. eutropha* H16. **a** Overexpression of RubL and RubS subunits from *R. eutropha* H16; **b** Overexpression of RubL, RubS subunits and GroES/GroEL from *R. eutropha*; **c** Overexpression of RubL, RubS subunits from *R. eutropha* and GroES/GroEL subunits from *E. coli*; **d** Overexpression of RubL, RubS and RubX from *Synechococcus* sp. PCC 7002; **e** Overexpression of RubL, RubS and RubX subunits from *Synechococcus* and GroES/GroEL subunits from *R. eutropha*; **f** Overexpression of RubL, RubS and RubX subunits from *Synechococcus* and GroES/GroEL subunits from *E. coli*; H16(pBBR1-RFP) is the control strain for each experiment. The values and error bars represent the means and SD of triplicate experiments

Overexpression plasmids with different combinations of RuBisCO genes and chaperone systems were constructed based on the pBBR1-MCS multiple-copy vector as listed in Additional file 1: Table S1. The strains carrying these plasmids were subjected for autotrophic gas fermentation in minimal medium supplemented with the gas mixture as described above. However, we found that most of the engineered strains had decreased growth compared with the control strain carrying an *rfp* expression plasmid with the same vector backbone (Fig. 2). The strains with decreased growth included those overexpressing only RuBisCO genes, RuBisCO genes together with the *E. coli* chaperone genes *groES/groEL*, and the endogenous *R. eutropha* RuBisCO together with endogenous *groES/groEL*. Only the strain H16(pRub_cyano, pGroESL_R), which overexpresses the *Synechococcus* sp. PCC 7002 RuBisCO genes together with the endogenous chaperone genes *groES/groEL* showed an increased growth phenotype. Its OD_600_ after 72 h of growth was 89.2% higher than that of the control strain. However, when fermentation was processed through 96 h, the difference between engineered and control strain was reduced (Fig. 2e). The engineered strain had a shorter lag phase probably due to the un-regulated expression of.

These results indicated that the assembly and maturation of a functional cyanobacterial RuBisCO in *R. eutropha* was successfully achieved with the assistance of overexpressed endogenous GroES/EL chaperons, as well as confirming the feasibility of increasing the carbon fixation efficiency of *R. eutropha* using heterologous RuBisCO enzymes.

### Engineering of the hydrogenase module and CBB cycle for improved autotrophic growth of *R. eutropha* C5

**Fig. 3:**
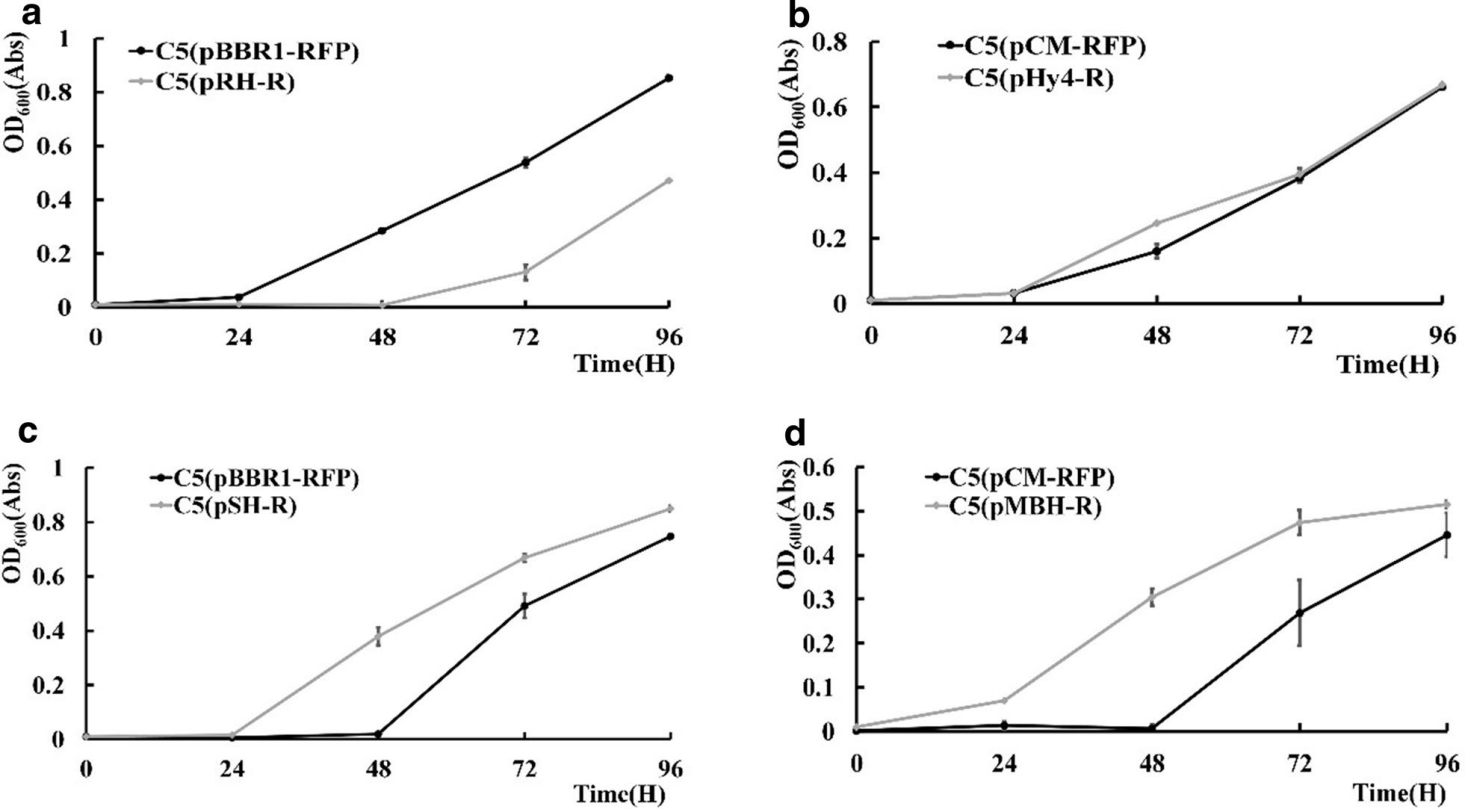
Autotrophic growth status of *R. eutropha* C5 strains overexpressing RH, SH, MBH or Hy4 hydrogenase. **a** RH structure subunits; **b** Hy4 subunits; **c** SH subunits; **d** MBH subunits; C5(pBBR1-RFP) is the control strain for each experiment. The values and error bars represent the means and SD of triplicate experiments

To engineer the hydrogenase systems, the genes encoding each hydrogenase were overexpressed using the same plasmids that were constructed for complementation in the knockout experiments, and the engineered strains were analyzed for their autotrophic growth phenotype. As illustrated in Fig. 3, while overexpression of the *hoxABCJ* genes encoding the RH hydrogenase decreased the growth (Fig. 3a), overexpressing the *PHG064* and *PHG065* genes encoding Hy4 had no effect. By contrast, expression of either *hoxFUYHI* encoding SH or *hoxKGZ* encoding MBH had a positive effect on the autotrophic growth. Compared with the control, after 96 h of autotrophic growth, the OD_600_ of the SH overexpression strain C5(pRH_R) and MBH overexpression strain C5(pMBH_R) increased 13.8 and 58.7%, respectively.

**Fig. 4:**
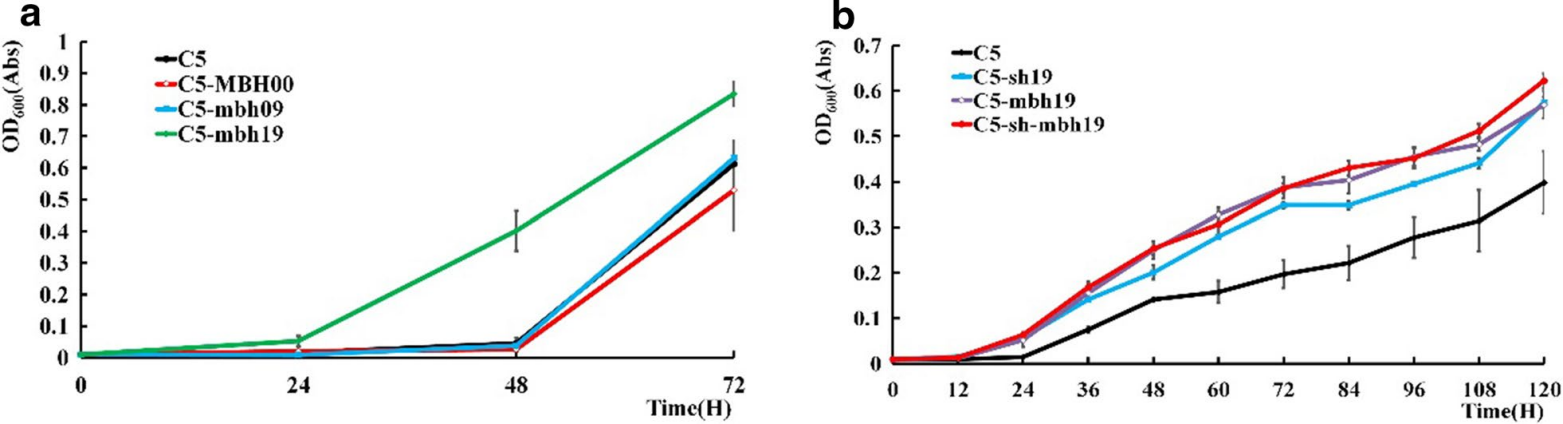
Autotrophic growth status of *R. eutropha* C5 strains with chromosomally upregulated expression of SH and MBH hydrogenases. **a** Promoter modification of the MBH gene cluster by replacing the native promoter with artificial promoters of different intensity; **b** promoter modulation of the MBH gene cluster, the SH gene cluster, or both SH gene clusters simultaneously. C5 is the control strain for each experiment. The values and error bars represent the means and SD of triplicate experiments

Since the plasmid expression system has limitations for more complex engineering, we intended to engineer the hydrogenase systems by modification the *R. eutropha* genome. The BBaJ_23100, BBaJ_23109 and BBaJ_23119 promoters from *E. coli* were selected (Additional file 1: Table S3) to modulate the expression of the MBH and SH hydrogenase operons. The corresponding strains were constructed by replacing the original promoters with the BBaJ promoters. Because the *R. eutropha* C5 strain we constructed is simpler to transform [24, 25], we decided to use it instead of H16 for these experiments. As shown in Fig. 4a, while the promoters BBaJ_23100 and BBaJ_23109 failed to increase the autotrophic growth, BBaJ_23119 with a stronger efficiency was able to increase the growth when inserted instead of the original MBH promoter. This effective regulator was introduced to increase the expression of the SH operon to obtain the strain C5-sh19. Subsequently, strain C5-shmbh19 in which both the MBH and SH operons were upregulated by BBaJ_23119 were constructed. Both strains C5-sh19 and C5-sh-mbh19 were found to have increased autotrophic growth compared with the parent strain, and C5-shmbh19 had a slightly higher growth than C5-sh19.

In this part, we modulated the expression of both SH and MBH by both plasmid-based overexpression and chromosomal promoter modulation. The results suggested that increased expression of both hydrogenase operons benefited the autotrophic growth of *R. eutropha* (Fig. 4b).

### Engineering of both the CBB module and the hydrogenase module improved the autotrophic growth and PHB production of *R. eutropha*

The genomic engineering of the hydrogenase operons provided a basis for engineering a strain in which both the CBB module and the hydrogenase module were engineered. By transforming strain C5-sh-mbh19 with the plasmids pRub_cyano and pGroESL_R, the strain C5-sh-mbh19(pRub_cyano, pGroESL_R) was obtained. Combining all the findings of this research, this strain may represent the most deeply engineered *R. eutropha* strain to date in terms of its autotrophic metabolism.

**Fig. 5:**
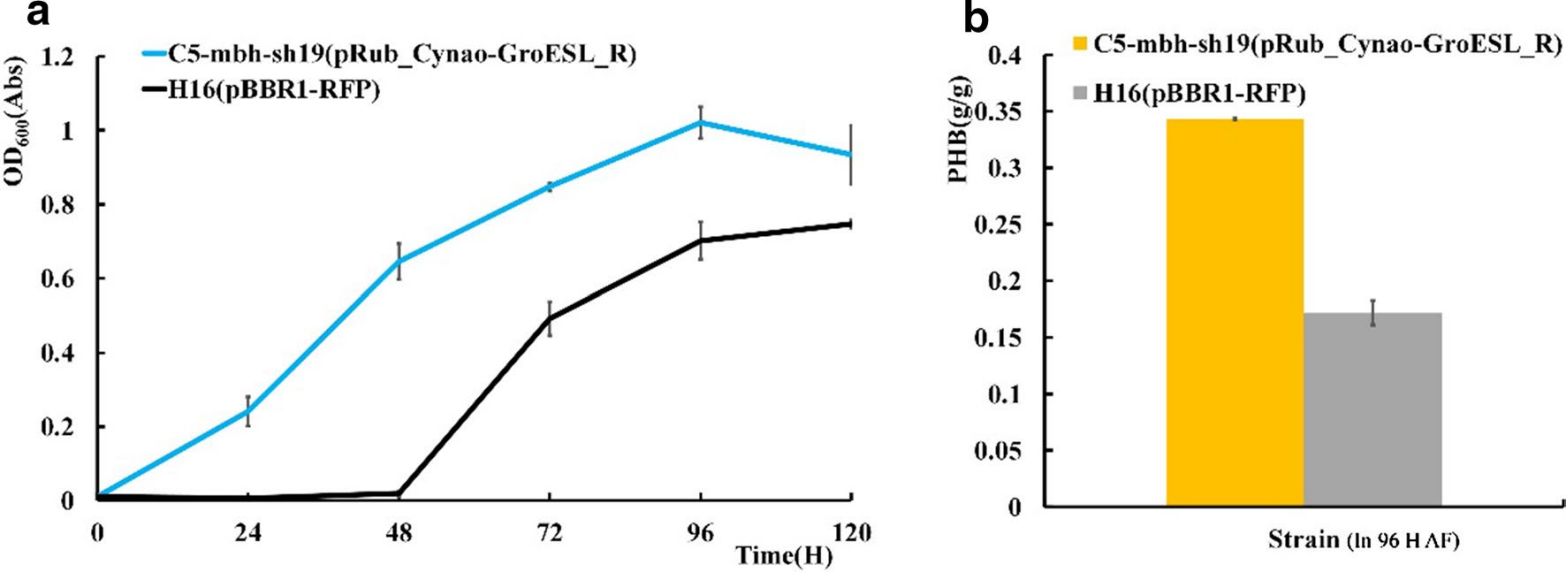
Growth and PHB production time course of the highly engineered strain C5-sh-mbh19(pRub_cyano, pGroESL_R) and the control strain in minimal medium with gas supplementation. **a** Growth curve of strain C5-sh-mbh19(pRub_cyano, pGroESL_R) and the control strain. **b** PHB production of strain C5-sh-mbh19(pRub_cyano, pGroESL_R) and the control strain. In 96 H AF: PHB production strain is in 96 h(H) autotrophic fermentation. The values and error bars represent the means and SD of triplicate experiments

*Ralstonia eutropha* can accumulate polyhydroxybutyrate (PHB) [26], which is a potential bioplastic material of great interest, in intracellular granules. Therefore, both the autotrophic growth status and PHB production capacity of C5-sh-mbh19(pRub_cyano, pGroESL_R) were evaluated. As illustrated in Fig. 5, the engineered strain displayed a substantial improvement in both the growth phenotype and PHB production, with increases of 93.4% and 74.7% in 96 h respectively compared to the parent strain. C5-mbh-sh19(pRub_cyano, pGroESL_R) produced 0.34 g/g PHB, which was a significant improvement over the 0.17 g/g produced by H16(pBAD-RFP).

The reason for selection of C5 strain as the parent strain in this work is due to the heavy genetic manipulation work requirement for engineering the autotrophic system of *R. eutropha*. The C5 strain provided a more convenient experimental subject for this work, which was constructed in our lab by knocking out only two putative restriction endonuclease genes H16_A0006 and H16_A0008-9 [25]. Since these two genes are by no means related to any autotrophic process, therefore we consider that C5 should perform the same with the wild type H16 strain, in terms of the metabolic manipulation towards the autotrophic system.

## Conclusions

The introduction of heterologous RuBisCO with higher efficiency into *R. eutropha* H16 coupled with overexpression of the endogenous GroES/EL chaperones was able to increase its autotrophic growth efficiency. At the same time, we found that both the MBH and SH hydrogenases contribute to the energy supply for autotrophic growth. We screened out a strong promoter from *E. coli*, BBaJ_23119, which could improve the expression of the chromosomal SH and MBH hydrogenase operons and increase the autotrophic growth. Finally, modification of both the CCB and hydrogenase modules was combined in one deeply engineered strain. With supplementation of only H_2_, CO_2_, and O_2_, both the autotrophic growth and PHB production were significantly increased. Although there might be other limiting factors, RuBisCO carboxylation efficiency is one of the key rate limiting reaction that restricts the CBB cycle and determines the plant carbon fixation efficiency and crop yield [27]. Increasing the efficiency of the RuBisCO system is one of the possible directions for significantly improving crop production. The successful optimization of autotrophic systems in bacteria provides an alternative and probably a better platform for the study and future improvement of carbon assimilation to increase crop yields.

## Methods

### Strains and culture conditions

The strains and plasmids used in this work are listed in Additional file 1: Table S1. *E. coli* S17 was used as the intermediate host for conjugational transfer. It was cultured at 37 °C and 250 rpm in lysogeny broth (LB) with appropriate antibiotics. *R. eutropha* H16 was grown at 30 °C and 250 rpm in LB or minimum medium with appropriate antibiotics. Antibiotic concentrations were 10 mg/L for gentamicin, 200 mg/L for kanamycin, and 30 mg/L for chloramphenicol. Plates were prepared by adding 1.5% agar to the liquid medium. Minimal medium contained 5.226 g/L NaH_2_PO_4_·2H_2_O, 11.55 g/L Na_2_HPO_4_·12H_2_O, and 0.453 g/L K_2_SO_4_, pH: 6.8–7.0.

### Plasmid construction

All plasmids and DNA oligo primes were designed using j5 DeviceEditor or Clonemanager [28]. All plasmids were assembled using Gibson assembly or CPEC [29, 30]. DNA segments were PCR-amplified using PrimeSTAR or Phusion High fidelity DNA polymerase (Takara, Japan). All DNA segments were purified using the SanPrep Column DNA Gel Extraction Kit (Sangon Biotech, China) before assembly. All plasmids were sequenced by Genewiz corporation (China) and all primes used in this study are listed in Additional file 1: Table S2.

### Preparation of competent cells and electro-transformation

Competent cells of *E. coli* S17 and *R. eutropha* H16 were prepared as described elsewhere [24, 25]. For *E. coli* transformation, we used pre-chilled sterile 1-mm gap electroporation cuvettes. After electroshock at 1.8 kV, 1 mL LB was added ant the cell suspension transferred to a 1.5 mL centrifuge and incubated at 37 °C and 250 rpm for 1 h for regeneration, after which the cells were spread on LB agar plates with the appropriate antibiotics. For *R. eutropha* transformation, we used 2-mm gap width cuvettes with an electric pulse of 2.3 kV, and regenerated the cells for 2 h in a 30 °C incubator.

### Gas fermentation

A batch fermentation system and a continuous fermentation system were used to analyze the cell growth status and PHB synthesis, respectively. The gas mixture used for autotrophic fermentation was composed of H_2_, CO_2_ and O_2_ at a ratio of 7:1:1. The H_2_ was supplied by a generator, and the CO_2_ and O_2_ were provided from gas tanks. The discontinuous fermentation system was composed of 100 mL headspace bottles with 10 mL minimal medium. For batch fermentation, the initial OD_600_ of *R. eutropha* was set to 0.01, after which the fermentation bottles were sparged with the gas mixture for 3 min to fill the head space. During the fermentation, the headspace was sparged with fresh gas mixture every 12 h and the bottles were incubated at 30 °C. For the continuous fermentation system, a 500 mL bioreactor (Additional file 1: Figure S1) with 150 mL of minimal medium was used. The gas mixture was continuously sparged into the fermenter at a flow rate of 60–70 mL/min. The continuous fermentation was conducted at 30 °C.

### Extraction and GC analysis of the PHB monomer 3-hydroxybutyric acid

After autotrophic fermentation, *R. eutropha* H16 cells were centrifuged at 8000 rpm for 5 min, washed twice with ddH_2_O, and put in a 70 °C oven for drying. The dry cells were weighed in a screw-capped glass tube, suspended in 2 mL methanol with 3% sulfuric acid and 2 mL chloroform, and heated at 100 °C for 4 h to achieve methyl esterification. After the sample was cooled, 1 mL of distilled water was added and vortexed for 5 min. The lower organic phase after static stratification was used for GC analysis after organic membrane filtration [31].

An Agilent 7890B gas chromatography system (Agilent, USA) equipped with an HP-5 column (Agilent, USA) was used for analysis. The GC program consisted of two stages. The first stage had an initial temperature of 80 °C and a final temperature of 140 °C. The initial hold time was 1.5 min and the rate of temperature increase was 30 °C/min. The second stage had an initial temperature of 140 °C and a final temperature of 220 °C. The hold time was 4.5 min and the rate of temperature increase was 40 °C/min. N_2_ was used as carrier gas and 3-hydroxybutyric acid standard (Sigma-Aldrich, USA) was used for quantification of the cellular PHB content. The standard GC curve of PHB is shown in supplemental Fig. 2.

## Supplementary information

**Additional file 1.** Supplement figures and tables.

## Data Availability

We provide supporting and necessary data for publication of the article. All supporting data is present in the article and the supplemental material documents. The strains and plasmids associated with this work will be physically available upon request from the authors.
