# Supplementary material for: Engineering the Calvin–Benson–Bassham cycle and hydrogen utilization pathway of Ralstonia eutropha for improved autotrophic growth and polyhydroxybutyrate production
Source: Microb Cell Fact. 2020 Dec 11;19:228. doi: 10.1186/s12934-020-01494-y (PMC7733298; doi:10.1186/s12934-020-01494-y)
Supplement: Supplementary file 1 — Additional file 1. Supplement figures and tables. [file 12934_2020_1494_MOESM1_ESM.docx]

**Additional file**

**Engineering the CBB cycle and hydrogen utilization pathway of *Ralstonia eutropha* for improved autotrophic growth and PHB production**

Zhongkang Li, Xiuqing Xin, Bin Xiong, Dongdong Zhao, Xueli Zhang & Changhao Bi

**Table S1. Strains and plasmid used in this study**

|  | **characteristics** | **references** |
| --- | --- | --- |
| *E.coli* |  |  |
| S17 | Host strain for transconjugation,  thi pro recA hsdR [RP4-2Tc::MuKm::Tn7] Tp^r^ Sm^r^ | Laboratory stock |
| *R. eutropha* |  |  |
| H16 | Wild type, Gen^r^ | ATCC 17669 |
| C5 | H16∆H16_A0006∆H16_A0008-9 | Laboratory stock |
| H16(pRrub) | pBBR1-MCS with rubisco units of *R. eutropha* | This study |
| H16(pRrub-EgroESL) | pBBR1-MCS with rubisco units of *R. eutropha* and GroESL of *E.coli* | This study |
| H16(pRrub-RgroESL) | pBBR1-MCS with rubisco units of *R. eutropha* and GroESL of *R.eutropha* | This study |
| H16(pCrub) | pBBR1-MCS with rubisco units of  *Synechococcus sp. PCC 7002* | This study |
| H16(pCrub-RgroESL) | pBBR1-MCS with rubisco units of  *Synechococcus sp. PCC 7002* and GroESL of *R. eutropha* | This study |
| H16(pCrub-EgroESL) | pBBR1-MCS with rubisco units of  *Synechococcus sp. PCC 7002* and GroESL of *E.coli* | This study |
| C5(pMBH-R) | pCM-MCS with MBH hydrogenase of *R.eutropha* | This study |
| C5(pSH-R) | pBBR1-MCS with SH hydrogenase of *R.eutropha* | This study |
| C5(pRH-R) | pBBR1-MCS with RH hydrogenase of *R.eutropha* | This study |
| C5(pHy4-R) | pCM-MCS with Hy4 hydrogenase of *R.eutropha* | This study |
| C5-mbh00 | BBa_J23100 promoter modification of C5 MBH gene cluster | This study |
| C5-mbh09 | BBa_J23109 promoter modification of C5 MBH gene cluster | This study |
| C5-mbh19 | BBa_J23119 promoter modification of C5 MBH gene cluster | This study |
| C5-sh19 | BBa_J23119 promoter modification of C5 MBH gene cluster | This study |
| C5-sh-mbh19 | BBa_J23119 promoter modification of C5 MBH gene cluster and SH gene cluster | This study |
| H16ΔRubC | Rubisco large and small units at chromosome 2 deletion of C5 | This study |
| H16ΔRubP | Rubisco large and small units at megaplasmid deletion of C5 | This study |
| H16ΔRubCΔRubP | Rubisco large and small units at chromosome 2 and megaplasmid deletion of C5 | This study |
| H16ΔMBH | HoxG,HoxK and HoxZ deletion of C5 | This study |
| H16ΔSH | HoxH,HoxY,HoxU and HoxF deletion of C5 | This study |
| H16ΔMBHΔSH | HoxG,HoxK, HoxZ HoxH,HoxY,HoxU and HoxF deletion of C5 | This study |
| C5-sh-mbh19(pRub_cyano, pGroESL_R) | BBa_J23119 promoter modification of C5 MBH gene cluster and SH gene cluster, pBBR1-MCS with rubisco units of  *Synechococcus sp. PCC 7002* and GroESL of *R. eutropha* | This study |

**Table S2. Primes used in this study**

| **Plasmids** | **Primes** | **Sequence(5’-3’)** |
| --- | --- | --- |
| pRrub | P_pBBR1_-F | CACACCAGGTCTCAGAGGATCCAAACTCGAGTAAGGATCTCCAGGC |
|  | P_pBBR1_-R | CACACCAGGTCTCATATGTATATCTCCTTCTTAAAGCTAGCACTGTACC |
|  | P_Rub_R_-F | CACACCAGGTCTCACATATGAACGCACCTGAATCGGTCCAAGC |
|  | P_Rub_R_-R | CACACCAGGTCTCACCTCAGTAGCGGCTGCCCTCGG |
| pRrub-EgroESL | P_pBBR1_-F | CACACCAGGTCTCAGCGGTGCTTAAGGATCCAAACTCG |
|  | P_pBBR1_-R | CACACCAGGTCTCATTCTCGCCATATGTATATCTCCTTCTTAAAGC |
|  | P_GroESL_R_-F | CACACCAGGTCTCAAGAACTCACTTTGTATCTAGGAGTCCG |
|  | P_GroESL_R_-R | CACACCAGGTCTCAAATAAGGGCAGTCGGTCGGC |
|  | P_Rub_R_-F | CACACCAGGTCTCATATTGCATCAACGCAGCAGCC |
|  | P_Rub_R_-R | CACACCAGGTCTCACCGCGCTCAGTAGCGGCTGCCCT |
| pRrub-EgroESL | P_pBBR1_-F | CACACCAGGTCTCAGCAGGATCCAAACTCGAGTAAGGATCTCCAGG |
|  | P_pBBR1_-R | CACACCAGGTCTCAGTATGTATATCTCCTTCTTAAAGCTAGCACTGTACC |
|  | P_GroESL_E_-F | CACACCAGGTCTCAATACAGATACGGACTTTCTCAAAGGAG |
|  | P_GroESL_E_-R | CACACCAGGTCTCACATCCAGTTCTACTGCGCCAG |
|  | P_Rub_R_-F | CACACCAGGTCTCAGATGCATCAACGCAGCAGCCAAGA |
|  | P_Rub_R_-R | CACACCAGGTCTCACTGCTCAGTAGCGGCTGCCC |
| pCrub_ | P_pBBR1_-F | CACACCAGGTCTCAAACTTAAGGATCCAAACTCGAGTAAGG |
|  | P_pBBR1_-R | CACACCAGGTCTCATATGTATATCTCCTTCTTAAAGCTAGCACTGTACC |
|  | P_RucL_cyano_-F | CACACCAGGTCTCACATATGGTTCAGACCAAATCTGCTGGG |
|  | P_RucL_cyano_-R | CACACCAGGTCTCACTCCTTCTTAAATTAGAGAGTGTCAACGGTATCGAATTCGAACT |
|  | P_RucXS_cyano_-F | CACACCAGGTCTCAGGAGATATACATATGGAGTTTAAAAAAGTTGCGAAGGAAACGGC |
|  | P_RucXS_cyano_-R | CACACCAGGTCTCAAGTTAGTAACGGGTTTGGTTGGGCTTGTAAACG |
| pCrub-EgroESL | P_pBBR1_-F | CACACCAGGTCTCAGCGGATCCAAACTCGAGTAAGGATCTCCAGGC |
|  | P_pBBR1_-R | CACACCAGGTCTCATATGTATATCTCCTTCTTAAAGCTAGCACTGTACC |
|  | P_GroESL_R_-F | CACACCAGGTCTCACATATGGTTCAGACCAAATCTGCTGGG |
|  | P_GroESL_R_-R | CACACCAGGTCTCAAACTCGAGTTTGGATCCTTAAGTTAGTAACGGGT |
|  | P_Rub_cyano_-F | CACACCAGGTCTCAAGTTGACGGCTAGCTCAGTCCTAGG |
|  | P_Rub_cyano_-R | CACACCAGGTCTCACCGCGTTGATGCAATAAGGGCAGTCGG |
| pCrub-EgroESL | P_pBBR1_-F | CACACCAGGTCTCAGAGGATCCAAACTCGAGTAAGGATCTCCAGGC |
|  | P_pBBR1_-R | CACACCAGGTCTCATATGTATATCTCCTTCTTAAAGCTAGCACTGTACC |
|  | P_GroESL_E_-F | CACACCAGGTCTCACATATGGTTCAGACCAAATCTGCTGGG |
|  | P_GroESL_E_-R | CACACCAGGTCTCAAACTCGAGTTTGGATCCTTAAGTTAGTAACGGGT |
|  | P_Rub_cyano_-F | CACACCAGGTCTCAAGTTGACGGCTAGCTCAGTCCTAGG |
|  | P_Rub_cyano_-R | CACACCAGGTCTCACCTCCAGTTCTACTGCGCCAGTTTCCG |
| pMBH_R | P_pCM_-F | CGATCGCAGGCTTTCCAAACGGATCCAAACTCG |
|  | P_pCM_-R | GCGCCAACCGGGGAGTATATCTCCTTCTTAAAGCTAGCACTGTACC |
|  | P_MBH_R_-F | GCTTTAAGAAGGAGATATACTCCCCGGTTGGCGCATCG |
|  | P_MBH_R_-R | GGATCCGTTTGGAAAGCCTGCGATCGCTTCGG |
| pSH_R | P_pBBR1_-F | CGGGGTTAGTCACCCAAACTCGAGTAAGGATCTCCAGGC |
|  | P_pBBR1_-R | CCTCCTTACTAATGTTCGATATCTCCTTCTTAAAGCTAGCACTGTACC |
|  | P_MBH_R_-F | AGCTTTAAGAAGGAGATATCGAACATTAGTAAGGAGGAGACAACATGG |
|  | P_MBH_R_-R | TACTCGAGTTTGGGTGACTAACCCCGTCCCCTCCC |
| pRH_R | P_pBBR1_-F | TGCGTGCCTGAGGGGATCCAAACTCGAGTAAGGATCTCCAGGC |
|  | P_pBBR1_-R | TGAGGCAAGACCGGTACATACAGATGTATATCTCCTTCTTAAAGCTAGCACTGTACC |
|  | P_RH_R_-F | GCTTTAAGAAGGAGATATACATCTGTATGTACCGGTCTTGCCTCAGC |
|  | P_RH_R_-R | CGAGTTTGGATCCCCTCAGGCACGCACTTCGC |
| pHy4-R | P_pCM_-F | CTCGCGAGGGAACTGAAGGATCCAAACGGATCCAAACTCG |
|  | P_pCM_-R | CTCAATGACCCGTGGATGTATATCTCCTTCTTAAAGCTAGCACTGTACCT |
|  | P_Hy4_R_-F | GAAGGAGATATACATCCACGGGTCATTGAGAGTCAAAGAACGC |
|  | P_Hy4_R_-R | GTTTGGATCCTTCAGTTCCCTCGCGAGTGCGC |
| pMBH100-R | P_pk18mobsacB_-F | CCAAGGATGCCAAACGAGCCGGAAGCATAAAGTGTAAAGCC |
|  | P_pk18mobsacB_-R | CTACTGCAGACCATTTCTTGCCGCCAAGGATCTGATGGC |
|  | P_MBH_L_-F | CCTTGGCGGCAAGAAATGGTCTGCAGTAGCTGCGTGGC |
|  | P_MBH_L_-R | GCACTGTACCTAGGACTGAGCTAGCCGTCAAGGGGAGCGATACCGCCCTCG |
|  | P_BBaJ23100_MBH_R_-F | CGGCTAGCTCAGTCCTAGGTACAGTGCTAGCGGTTGGCGCATCGCGACGA |
|  | P_BBaJ23100MBH_R_-R | GCTTCCGGCTCGTTTGGCATCCTTGGCCACGT |
| pMBH109-R | P_pk18mobsacB_-F | CCAAGGATGCCAAACGAGCCGGAAGCATAAAGTGTAAAGCC |
|  | P_pk18mobsacB_-R | ACTGCAGACCATTTCTTGCCGCCAAGGATCTGATGGC |
|  | P_MBH_L_-F | CCTTGGCGGCAAGAAATGGTCTGCAGTAGCTGCGTGGC |
|  | P_MBH_L_-R | GCACAGTCCCTAGGACTGAGCTAGCTGTAAAGGGGAGCGATACCGCCCTCG |
|  | P_BBaJ23109_MBH_R_-F | TTACAGCTAGCTCAGTCCTAGGGACTGTGCTAGCGGTTGGCGCATCGCGACG |
|  | P_BBaJ23109_MBH_R_-R | GCTTCCGGCTCGTTTGGCATCCTTGGCCACGT |
| pMBH119-R | P_pk18mobsacB_-F | CCAAGGATGCCAAACGAGCCGGAAGCATAAAGTGTAAAGCC |
|  | P_pk18mobsacB_-R | CTACTGCAGACCATTTCTTGCCGCCAAGGATCTGATGGC |
|  | P_MBH_L_-F | CCTTGGCGGCAAGAAATGGTCTGCAGTAGCTGCGTGGC |
|  | P_MBH_L_-R | GCATTATACCTAGGACTGAGCTAGCTGTCAAGGGGAGCGATACCGCCCTCG |
|  | P_BBaJ23119_MBH_R_-F | CCCCTTGACAGCTAGCTCAGTCCTAGGTATAATGCTAGCGGTTGGCGCATCGCGACGA |
|  | P_BBaJ23119_MBH_R_-R | GCTTCCGGCTCGTTTGGCATCCTTGGCCACGT |
| pSH119-R | P_pk18mobsacB_-F | CACACCAGGTCTCAGACTCACATTAATTGCGTTGCG |
|  | P_pk18mobsacB_-R | CACACCAGGTCTCAGGTGCTAAAGGAAGCGGAACACG |
|  | P_SH_L_-F | CACACCAGGTCTCACACCCGAAGTGCCAGTCGCTCG |
|  | P_SH_L_-R | CACACCAGGTCTCAAGGACTGAGCTAGCTGTCAATGCTTGGTGCGTCACTTCG |
|  | P_BBaJ23119_SH_R_-F | CACACCAGGTCTCATCCTAGGTATAATGCTAGCAGGGGCGAACATTAGTAAGGAGG |
|  | P_BBaJ23119_SH_R_-R | CACACCAGGTCTCAAGTCCGGCCACGGAAGAACACCG |
| pΔMBH | P_pk18mobsacB_-F | ACCTTTAAGGACTGCCTGGGGTGCCTAATGAGTGAGC |
|  | P_pk18mobsacB_-R | TCGCGAAATGCCCCGCGATAAGCTAGCTTCACGCTGC |
|  | P_MBH_L_-F | CTAGCTTATCGCGGGGCATTTCGCGACGAAGTTTCC |
|  | P_MBH_L_-R | TGCAATGCGATCCGCCCATCTTGTATAAGCAGAAGCCTTTGCGGGC |
|  | P_MBH_R_-F | TCTGCTTATACAAGATGGGCGGATCGCATTGCAGATGCG |
|  | P_MBH_R_-R | CATTAGGCACCCCAGGCAGTCCTTAAAGGTCCGATAGCCCG |
| pΔSH | P_pk18mobsacB_-F | CACACCAGGTCTCATGACATTAATGAATCGGCCAACGCGCGG |
|  | P_pk18mobsacB_-R | CACACCAGGTCTCAATAGCTACTGGGCTATCTGGACAAGGG |
|  | P_SH_L_-F | CACACCAGGTCTCACTATGGATAGTCGTATCACGACAATACTCGAGCG |
|  | P_SH_L_-R | CACACCAGGTCTCAGTGTGAATACCACCTTATCGATCAGCATCGCC |
|  | P_SH_R_-F | CACACCAGGTCTCAACACGGATTGGGGTGGGCTTTCATCG |
|  | P_SH_R_-R | CACACCAGGTCTCAGTCACGAGGTTTGACGCTCGGCC |
| pΔRuP | P_pk18mobsacB_-F | GGAATTGCACACACCGGCCTGGGGTGCCTAATGAGTGAGC |
|  | P_pk18mobsacB_-R | GCAATGCGCTGGACGATAAGCTAGCTTCACGCTGCCGC |
|  | P_RuP_L_-F | GTGAAGCTAGCTTATCGTCCAGCGCATTGCGCACGC |
|  | P_RuP_L_-R | CGGCGACTACGAGGAGAGCTACGCCGTGCAGGCG |
|  | P_RuP_R_-F | CGGCGTAGCTCTCCTCGTAGTCGCCGTCCCAGTACCC |
|  | P_RuP_R_-R | CATTAGGCACCCCAGGCCGGTGTGTGCAATTCCAGCG |
| pΔRuC | P_pk18mobsacB_-F | CGTTACACGATGCAAAGCCTGGGGTGCCTAATGAGTGAGC |
|  | P_pk18mobsacB_-R | CCCGGGCATGTCCCGCGATAAGCTAGCTTCACGCTGC |
|  | P_RuC_L_-F | CTAGCTTATCGCGGGACATGCCCGGGTTGGACTCG |
|  | P_RuC_L_-R | CGGCGACTACGAGAGCTACGCTGTGCAGGCAC |
|  | P_RuC_R_-F | GCACAGCGTAGCTCTCGTAGTCGCCGTCCCAGTACCC |
|  | P_RuC_R_-R | CATTAGGCACCCCAGGCTTTGCATCGTGTAACGGGTGCC |

**Table S3.** **Genes and the coded enzyme of CBB operon of *Ralstonia eutropha***

| **Gene** | **Coded enzyme** |
| --- | --- |
| **R** | LysR-type transcriptional activator binding to the operator of the cbb control region |
| **L** | RuBisCO ribulose-1,5-bisphosphate carboxylase/oxygenase large subunits |
| **S** | RuBisCO ribulose-1,5-bisphosphate carboxylase/oxygenase small subunits |
| **X** | unknown |
| **Y** | unknown |
| **E** | pentose-5-phosphate 3-epimerase |
| **F** | fructose-1,6-/sedoheptulose-1,7-bisphosphatase |
| **P** | PRK phosphoribulokinase, |
| **T** | transketolase |
| **Z** | 2-phosphoglycolate phosphatase |
| **G** | glyceraldehyde-3-phosphate dehydrogenase |
| **K** | phosphoglycerate kinase |
| **A** | fructose-1,6-/sedoheptulose-1,7- bisphosphate aldolase |
| **B** | formate dehydrogenase (FDH)-like protein |


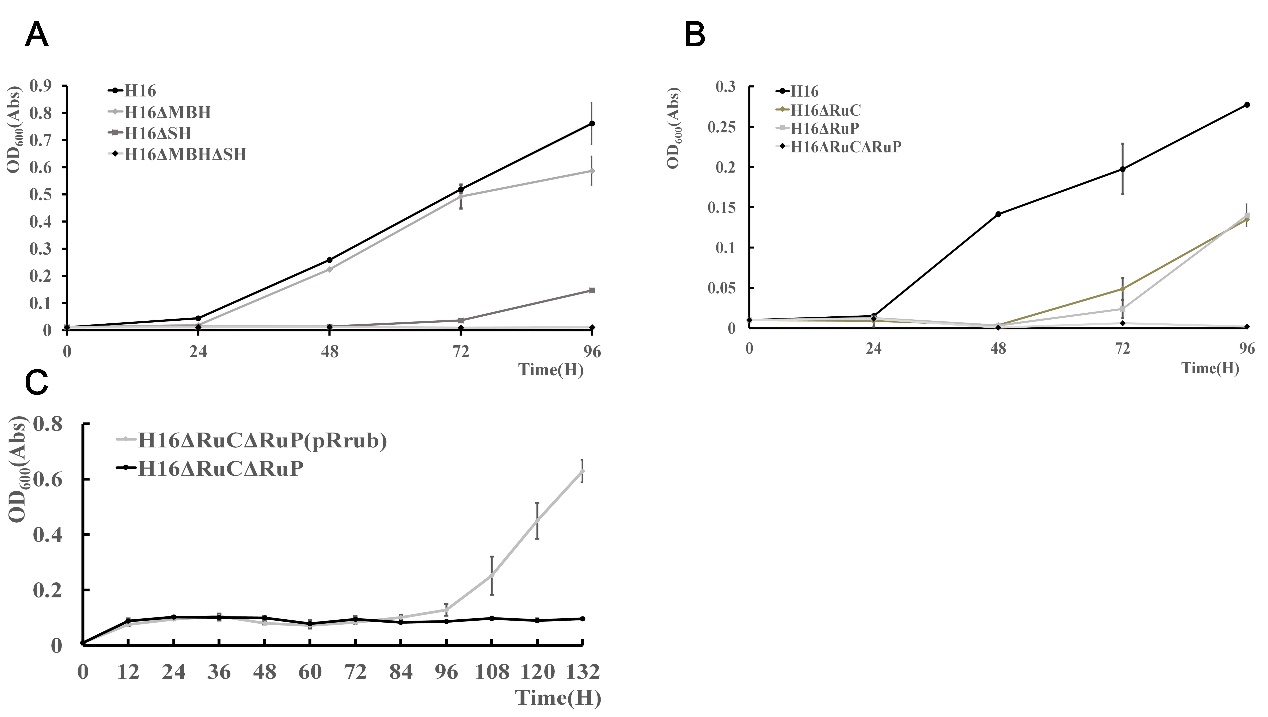


**Figure S1: Autotrophic growth status of *R. eutropha* H16 with deletions of the** **RuBisCO operons or the MBH and SH hydrogenase genes**

(A) Growth curves of the MBH and SH deletion strains; (B): Growth curves of RuP (RuBisCO operon on megaplasmid) or RuC (RuBisCO operon on chromosome 2) deletion strains. (C) Growth curve of the complemented RuBisCO double deletion strain. The values and error bars represent the means and SD of triplicate experiments. pRrub plasmid expresses rubisco units of *R. eutropha* (Table S1).


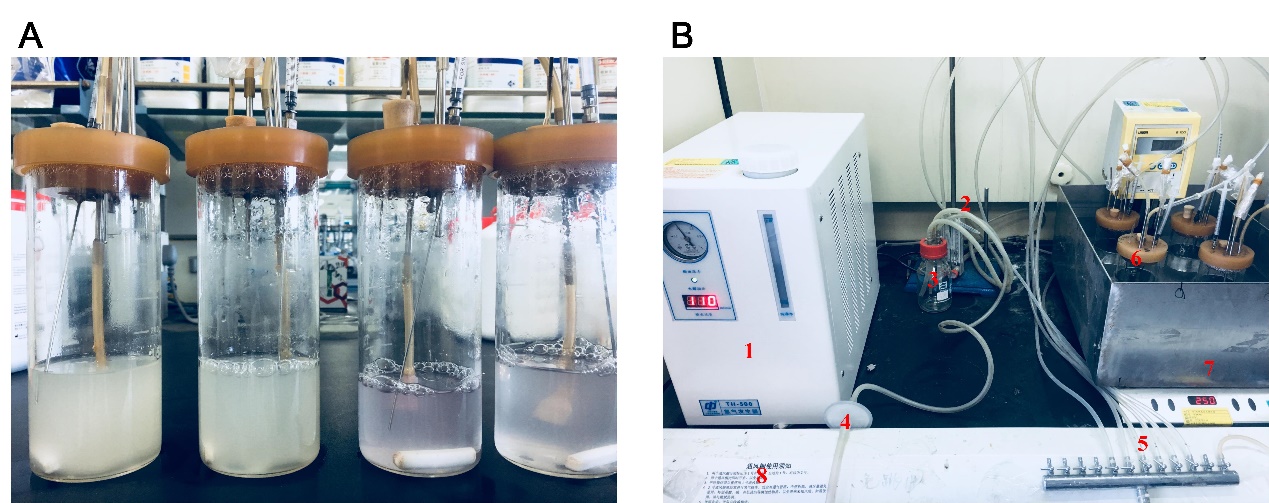


**Figure S2. Gas continuous fermentation system used in this study**

1: Hydrogen Generator; 2: Rotameter; 3: Mixed Gas Tank; 4: Filtrator; 5: Multi-hole Gas Shunt; 6: 500mL fermentation cylinder; 7: Thermostatic water bath magnetic Stirrer; 8: Fume hood.


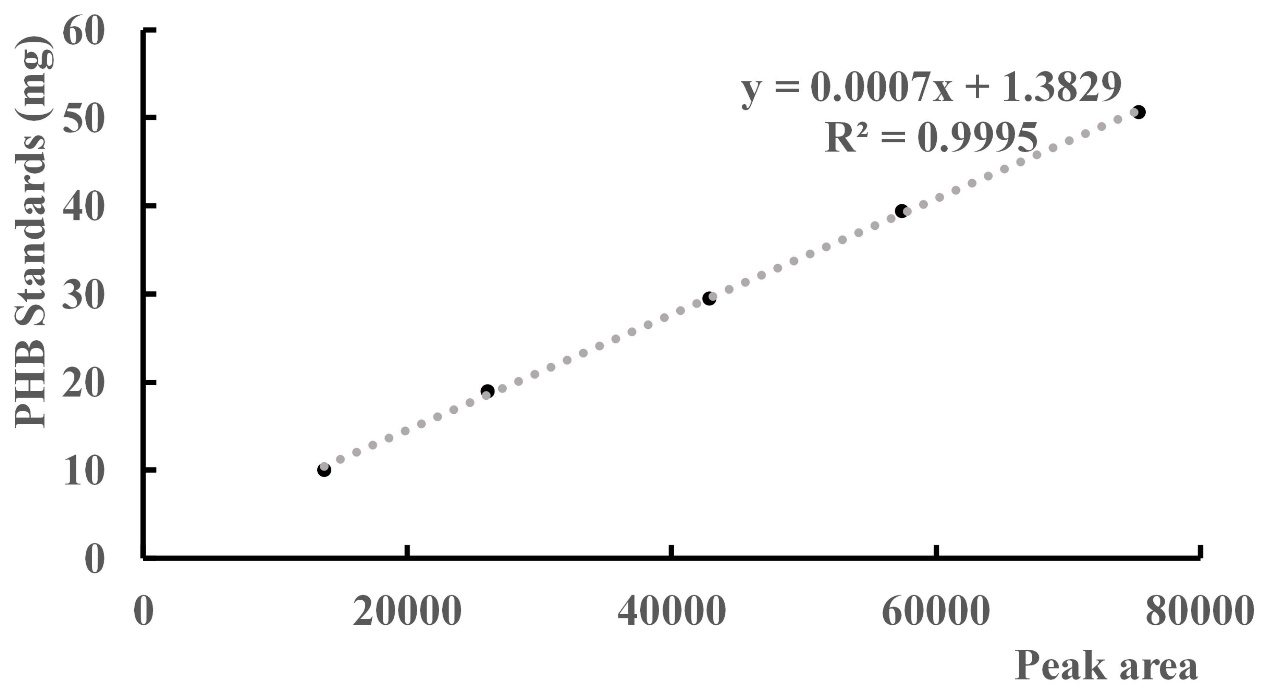


**Figure S3. The calibration curve of PHB standards gas chromatographic method**

**Table S4. Strengths of three kinds of promoter used in hydrogenase engineering**

| Promoter | Strength |
| --- | --- |
| BBa_J23109 | 106 |
| BBa_J23100 | 2547 |
| BBa_J23119 | Strongest(unkonw) |
